# Characterization of Maternal Circulating MicroRNAs in Obese Pregnancies and Gestational Diabetes Mellitus

**DOI:** 10.3390/antiox12020515

**Published:** 2023-02-17

**Authors:** Anaïs Serati, Chiara Novielli, Gaia Maria Anelli, Maria Mandalari, Francesca Parisi, Irene Cetin, Renata Paleari, Chiara Mandò

**Affiliations:** 1 Department of Biomedical and Clinical Sciences, Università degli Studi di Milano, 20157 Milan, Italy; 2 Department of Pathophysiology and Transplantation, Università degli Studi di Milano, 20054 Segrate, Italy; 3 Department of Woman, Mother and Neonate, Buzzi Children’s Hospital, ASST Fatebenefratelli Sacco, 20154 Milan, Italy

**Keywords:** pregnancy, maternal obesity, gestational diabetes mellitus, maternal epigenetics, miRNA, circulating miRNA

## Abstract

Maternal obesity (MO) is expanding worldwide, contributing to the onset of Gestational Diabetes Mellitus (GDM). MO and GDM are associated with adverse maternal and foetal outcomes, with short- and long-term complications. Growing evidence suggests that MO and GDM are characterized by epigenetic alterations contributing to the pathogenesis of metabolic diseases. In this pilot study, plasma microRNAs (miRNAs) of obese pregnant women with/without GDM were profiled at delivery. Nineteen women with spontaneous singleton pregnancies delivering by elective Caesarean section were enrolled: seven normal-weight (NW), six obese without comorbidities (OB/GDM(−)), and six obese with GDM (OB/GDM(+)). miRNA profiling with miRCURY LNA PCR Panel allowed the analysis of the 179 most expressed circulating miRNAs in humans. Data acquisition and statistics (GeneGlobe and SPSS software) and Pathway Enrichment Analysis (PEA) were performed. Data analysis highlighted patterns of significantly differentially expressed miRNAs between groups: OB/GDM(−) vs. NW: *n* = 4 miRNAs, OB/GDM(+) vs. NW: *n* = 1, and OB/GDM(+) vs. OB/GDM(−): *n* = 14. For each comparison, PEA revealed pathways associated with oxidative stress and inflammation, as well as with nutrients and hormones metabolism. Indeed, miRNAs analysis may help to shed light on the complex epigenetic network regulating metabolic pathways in both the mother and the foeto-placental unit. Future investigations are needed to deepen the pregnancy epigenetic landscape in MO and GDM.

## 1. Introduction

The hypothesis of the “Developmental Origin of Health and Disease” (DOHaD) or “foetal programming” postulates that pregnancy represents a crucial time frame not only for the development and growth of the foetus, but also for the establishment of the health of the future adult [1]. During gestation, the exposure to an altered maternal–foetal milieu and to adverse conditions, such as maternal malnutrition and metabolic alterations, can permanently affect the structure and the function of foetal organs and systems, which are characterized by high developmental plasticity [2]. Maternal obesity, in particular, is emerging as one of the main issues for short- and long-term health consequences for both the mother herself and the offspring. Indeed, it can affect pregnancy outcomes, enhancing the risks of preterm delivery, stillbirth, foetal malformations, and growth disturbances, including either intrauterine growth restriction or macrosomia. Moreover, both the mother and the baby are more susceptible to long-term complications such as obesity, type II diabetes, metabolic diseases, and cardiovascular pathologies later in life [3,4].

The worldwide incidence of obesity among pregnant women is estimated at 18.5–38.3%. Moreover, the risk of developing Gestational Diabetes Mellitus (GDM) increases up to 50% in obese women [5,6]. Maternal obesity and GDM are associated with systemic low-grade chronic inflammation and excessive increase in oxidative stress, altered release of adipokines (e.g., leptin) and cytokines (e.g., TNF-α, IL-6, IL-1β), and lipotoxicity. Together, these alterations result in a perturbed intrauterine environment affecting placental structure and function [3,7]. Placental dysfunction and perturbations of the intrauterine environment can also influence pregnancy outcomes and foetal health programming through epigenetic modifications, in particular microRNAs (miRNAs) [8,9,10]. Increasing evidence points out that miRNA regulation is fundamental to the establishment and maintenance of pregnancy and is implicated in the pathogenesis of gestational disorders [11]. Indeed, intrauterine exposures to an obesogenic environment can influence birth and foetal outcomes via microRNAs and alterations in their expression have been found in pregnancy-associated pathologies, including GDM [12,13,14].

During pregnancy, specific miRNAs are produced by the mother, the foetus, or the placenta. This pool of pregnancy-associated miRNAs can be resident in tissues or circulate freely. In fact, part of the pool is released into the blood flow, freely or packaged into extracellular vesicles, providing an effective means of communication between the three compartments of pregnancy [13]. miRNAs detected in maternal circulation may therefore reflect the status of the maternal–foetal interface and of the intrauterine environment, thus representing potential non-invasive biomarkers for pregnancy disorders [15]. 

Although few studies have been conducted to investigate pregnancy-related microRNAs [16,17,18,19,20], epigenetic characterization is still only partially complete, especially in obese and/or GDM pregnant women.

Therefore, the aim of this work is to enrich the current knowledge by studying the most frequently expressed circulating microRNAs in obese pregnant women, with or without GDM, in order to shed light on the possible alterations in metabolically impaired pregnancies.

## 2. Materials and Methods

### 2.1. Population

This observational pilot study involved a cohort of pregnant women enrolled at the Obstetric Units of “Luigi Sacco” and “Vittore Buzzi” Hospitals (ASST Fatebenefratelli-Sacco) in Milan, Italy. The study was conducted in accordance with the Declaration of Helsinki and in compliance with current Good Clinical Practice guidelines, local laws, regulations, and organizations. The protocol was approved by the Ethical Committee [Prot. n°17739/2018] and all participants gave their informed consent for personal data treatment and biological specimens collection.

We recruited 19 Caucasian pregnant women aged 18–42 years with single-term pregnancies at elective Caesarean Section (eCS). The study population was divided according to pre-pregnancy BMI (kg/m^2^), following the Institute of Medicine (IOM) guidelines [21], and to the presence of GDM, which was diagnosed according to the International Federation of Gynaecology and Obstetrics (FIGO) guidelines: Oral Glucose Tolerance Test (OGTT)-75 g was performed at 24–28 weeks [22,23]:

- Normal Weight (NW): 18 kg/m^2^ ≤ BMI < 25 kg/m^2^, *n* = 7;
- Obese without comorbidities (OB/GDM(−)): BMI ≥ 30 kg/m^2^, *n* = 6;
- Obese with GDM (OB/GDM(+)): BMI ≥ 30 kg/m^2^, *n* = 6.

OB women underwent regular checks and received specific diet and lifestyle counselling to avoid excessive Gestational Weight Gain (GWG), as recommended by IOM [21]:

- NW: 11.5 ≤ GWG ≤ 16 Kg;
- OB: 5 ≤ GWG ≤ 9 Kg.

With the sole exception of pregestational obesity and gestational diabetes, already defined as inclusion criteria, any other known maternal pregestational chronic disease or drug consumption, as well as smoking habit and alcohol or substance abuse, represented exclusion criteria. Furthermore, any other pregnancy-related diseases (i.e., hypertensive disorders) or GDM under pharmacological treatment were exclusion or drop-out criteria. 

Maternal and foetal data were recorded at delivery.

### 2.2. Maternal Plasma Collection

Immediately prior to eCS, maternal venous blood was collected from the cubital vein into EDTA tubes and centrifuged at 1500 rpm for 15 min at room temperature to obtain plasma. Aliquots were stored at −80 °C.

### 2.3. MicroRNA Profiling

Non-haemolysed maternal plasma samples were selected. Total RNA was extracted from 200 μL of plasma using the miRNeasy Serum/Plasma Advanced Kit, which allows retrieval of low-abundance miRNAs, in conjunction with the miRNeasy Serum/Plasma Spike-In Control cel-miR-39-3p. Next, 2 μL of the extracts were retrotranscribed into specific miRNA-based cDNA with a miRCURY LNA RT Kit, using the UniSp6 spike-in miRNA as retrotranscription control. 

Real-time PCR experiments were performed on Serum/Plasma-Focus PCR Panels (YAHS-106YC-8), ready-to-use plates with specific primers for the 179 most expressed miRNAs in human serum/plasma, with reagents from the miRCURY LNA SYBR Green PCR Kit.

All procedures were carried out according to the manufacturer’s instructions (Qiagen, German Town, MD, USA).

### 2.4. Data Analysis and Statistics

Maternal and foetal data were analysed with SPSS, v.27 (IBM, Armonk, NY, USA). Statistical significance was set for *p* < 0.05.

According to data distribution (Kolmogorov–Smirnov test), one-way ANOVA (parametric statistics with Tukey’s HSD as post hoc analysis), or Kruskal–Wallis tests (non-parametric statistics with Pairwise comparison as post hoc analysis) were performed to evaluate differences between NW, OB/GDM(−) and OB/GDM(+) groups, with Bonferroni correction.

For miRNA data analysis, raw Ct were obtained using ThermoFisher Cloud and then uploaded on GeneGlobe Data-Analysis Center (Qiagen), where project-specific settings were defined (i.e., experimental groups: OB/GDM(−) as Group 1, OB/GDM(+) as Group 2, NW as Control Group; internal controls: Cel-miR-39-3p as extraction control, UniSp6 as retrotranscription control, UniSp3 IPC as Inter Plate Calibrator). The lower limit of detection was set at 36 so that greater Ct values were not considered for further analysis. Data were normalized using NormFinder method; this algorithm attempts to find the optimum reference miRNAs out of a group of candidates, choosing the 10 most stably expressed miRNAs among all subjects (with standard deviation of Ct < 0.25) [24]. The software allows comparison between two groups. It calculates: average ∆Ct, 2^(−∆Ct)^, ∆∆Ct, 2^(−∆∆Ct)^ (i.e., Fold Change = X), and Fold Regulation (−1/X), referring to all three possible comparisons: (A) OB/GDM(−) (Case Group 1) vs. NW (Control Group), (B) OB/GDM(+) (Case Group 2) vs. NW (Control Group), or C) OB/GDM(+) (Case Group 2) vs. OB/GDM(−) (Case Group 1).

Fold Regulation cut-off was set for values >|2| (Fold Regulation >2 or <−2), meaning differential expression between groups of at least two times in positive (upregulation) or negative (downregulation).

An unpaired two-tailed *t*-Test was used to compare the 2^(−ΔCt)^ values for each miRNA in the two groups for each of the three comparisons. Statistical significance was set for a *p*-value < 0.05. 

For each of the three comparisons, miRNAs showing differential expression with a Fold Regulation >2 or <−2 and statistical significance (*p*-value < 0.05) were further investigated to better understand their biological role. Pathways prediction analyses were performed using two of the most updated and literature-accepted online bioinformatics tools: miTALOS and DIANA-miRPath, as previously described by our group [25]. 

## 3. Results

### 3.1. Clinical Characteristics of the Study Population

Table 1 summarizes maternal and foetal characteristics of the three study groups.

The three groups were homogeneous in terms of maternal age. In agreement with the inclusion criteria, pre-pregnancy BMI was significantly higher in both OB/GDM(−) and OB/GDM(+) vs. NW (*p* = 0.032 and *p* = 0.001, respectively), and all OGTT values tended to be higher in OB/GDM(+) vs. both the NW and OB/GDM(−) group, despite not reaching statistical significance (probably because of the groups’ small sizes). On average, all three groups did not show excessive GWG, according to IOM guidelines [21], and GWG tended to be lower in both obese groups vs. NW. On average, all women delivered at full term (gestational age > 39 weeks). Foetal characteristics did not differ among the three study groups.

### 3.2. MicroRNA Profiling in Maternal Plasma

The data quality control selected the results of 16 subjects (6 NW, 5 OB/GDM(−), 5 OB/GDM(+)), out of the 19 tested, for downstream investigation. Indeed, 3 subjects (1 NW, 1 OB/GDM(−), 1 OB/GDM(+)) were excluded because of excessively high normalized Ct value of the extraction control Cel-miR-39-3p compared to the other samples.

**Figure 1:**
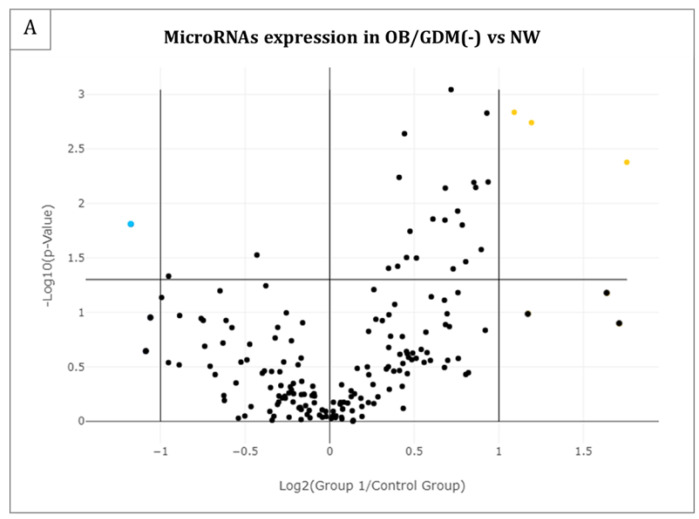

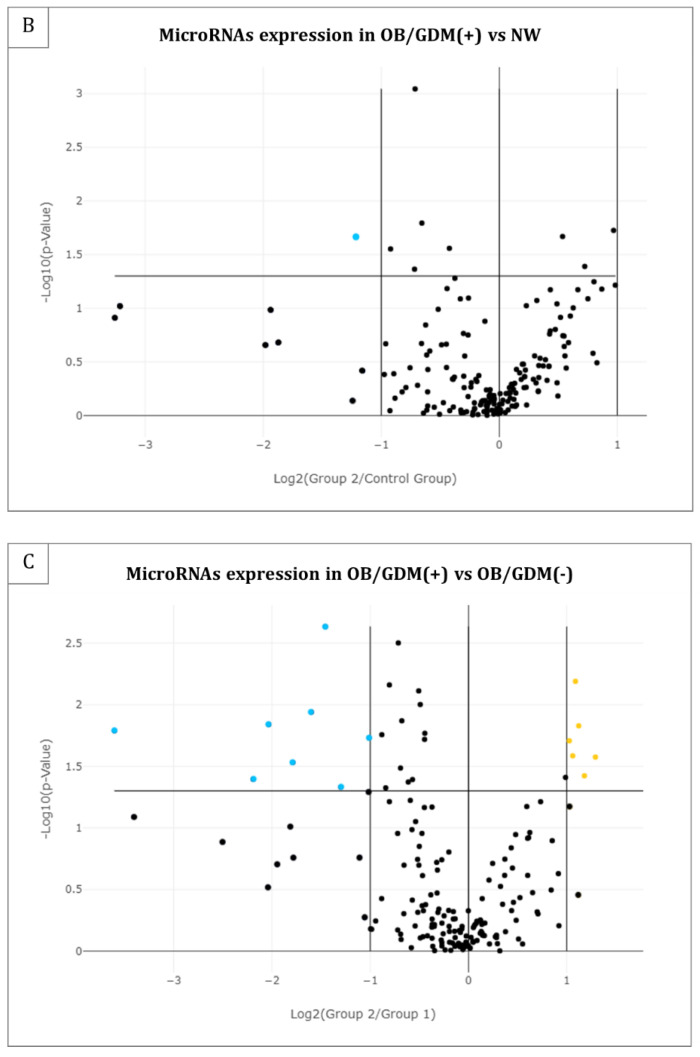
Volcano Plots of the 179 analysed microRNAs in maternal plasma for the following comparisons: (**A**) OB/GDM(−) vs. NW, (**B**) OB/GDM(+) vs. NW, and (**C**) OB/GDM(+) vs. OB/GDM(−). Significant miRNA expression changes for each group comparison are presented by plotting the log2 of the Fold Changes in miRNA expression on the x-axis versus their statistical significance on the y-axis. The two vertical lines represent Fold Regulation cut-off of |2|, while the horizontal line indicates the statistical significance (*p*-value) of 0.05. MiRNAs in the far upper left (downregulated: blue points) and far upper right (upregulated: yellow points) sections meet the selected fold regulation and *p*-value thresholds; the black points identify all the miRNAs which do not comply with these criteria. Data and graph obtained by GeneGlobe (Qiagen).

Table 2 lists the microRNAs that resulted differentially expressed in each of the three groups’ comparisons. Figure 1 reports the same results as Volcano Plots. In particular, *n* = 4 miRNAs resulted significantly differentially expressed (*p*-value < 0.05) and with a fold regulation > |2| in OB/GDM(−) vs. NW comparison (Table 2 and Figure 1A); *n* = 1 miRNA in OB/GDM(+) vs. NW (Table 2 and Figure 1B); *n* = 14 miRNAs in OB/GDM(+) vs. OB/GDM(−) (Table 2 and Figure 1C).

To gain insights into the miRNA profiling results, DIANA–miRPath and miTALOS online tools were used for miRNA pathway enrichment analysis.

First, each cluster of miRNAs that resulted significantly and differentially expressed between (A) OB/GDM(−) vs. NW, (B) OB/GDM(+) vs. NW, and (C) OB/GDM(+) vs. OB/GDM(−) was inserted in DIANA–miRPath v.3, and union analysis (i.e., all pathways significantly targeted by at least one miRNA out of the initially selected group are included) was performed. Table S1 shows the complete results of this analysis.

**Figure 2:**
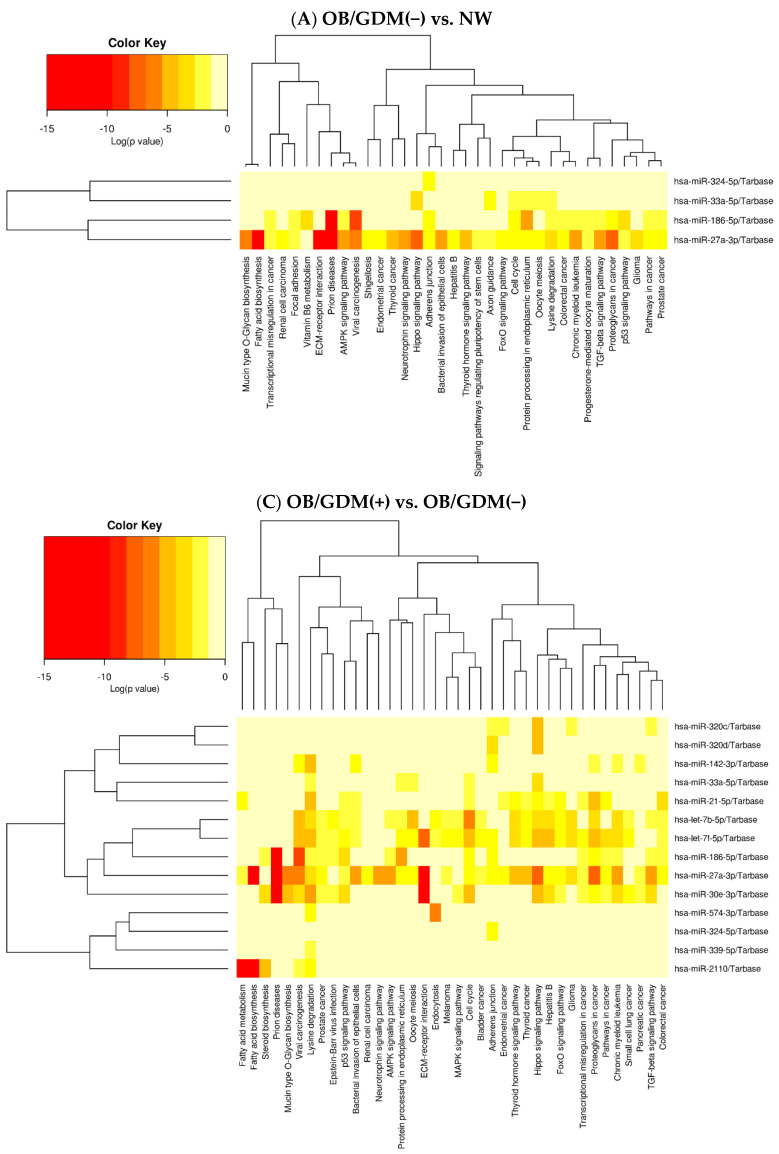
Heat maps by DIANA–miRPath v.3 showing miRNAs’ clusters and associated pathways obtained by union analysis for the following comparisons: (A) OB/GDM(−) vs. NW, and (C) OB/GDM(+) vs. OB/GDM(−). For each panel, the analysed miRNAs are listed on the right. The specific pathways, determined to be significant via miRNA pathway enrichment analysis, are listed at the bottom. The force of association (Log(*p*-value)) between the analysed miRNAs and the pathways is indicated by the colour key reported in the upper left corner. Log: log10 *p*-value.

Heat maps depicting the force of association between specific miRNAs’ cluster and related pathways were obtained for comparisons (A) and (C) (Figure 2A,C). It was not possible to obtain the Heat map for comparison (B) because it included only one miRNA (hsa-miR-454-3p).

From the complete results obtained via DIANA-miRPath union analysis, resumed in Table S1 and Figure 2, the most coherent pathways with the context of (maternal) obesity, diabetes/GDM, pregnancy, oxidative stress and inflammation were selected for further discussion. Consequently, pathways related to cancer, infectious diseases, or to ubiquitous mechanisms present in every eukaryotic cell type have been omitted in the following sections. The selected pathways, with related force of association (i.e., *p*-value) and specifically associated miRNAs, are reported in Table 3.

To complete our bioinformatic search, an additional pathway enrichment union analysis was also performed by miTALOS v.2, inserting the significantly differentially expressed miRNAs (Table 2). Relevant pathways are listed below:

(A) OB/GDM(−) vs. NW:

- SREBF (Sterol Regulatory Element-Binding transcription Factor) and miR33 in cholesterol and lipid homeostasis (*p* = 0.017981);
- Insulin signalling (*p* = 0.042847).

(B) OB/GDM(+) vs. NW:

- TGF-beta (Transforming Growth Factor-beta) signalling pathway (*p* = 0.005859);

(C) OB/GDM(+) vs. OB/GDM(−):

- mTOR (mammalian Target Of Rapamycin) signalling pathway (*p* = 0.044364),
- Insulin signalling (*p* = 0.013768).

## 4. Discussion

The aim of this pilot study was to profile maternal plasma miRNAs in a well-characterized cohort of normal weight and obese women affected or unaffected by GDM, at term of pregnancy, and to seek possible associations with a dysregulated maternal metabolic state. To our knowledge, this is the first study to simultaneously analyse a panel of 179 miRNAs in maternal plasma with a real-time-based method. MiRNAs presenting significantly different expression in the comparison between groups underwent a pathway enrichment analysis (PEA) using the bioinformatic tools DIANA-miRPath and miTALOS.

In the comparison (A) OB/GDM(−) vs. NW, four miRNAs were significantly differentially expressed. DIANA-miRPath showed a set of 35 signalling pathways specifically associated with this miRNAs’ pattern. The analysis (B) OB/GDM(+) vs. NW reported one differentially expressed miRNA associated with 15 molecular pathways. Finally, comparison (C) OB/GDM(+) vs. OB/GDM(−) showed 14 miRNAs significantly differentially expressed, associated with 38 pathways.

For each comparison, among the (A) 35, (B) 15, (C) 38 pathways, the most relevant ones in the context of pregnancy, maternal obesity, GDM and metabolic derangements were selected for further literature search and discussion (Table 3). An additional analysis with miTALOS was also performed, pointing out some supplementary potentially interesting pathways.

The main findings are discussed below, divided into several main topics.

### 4.1. Lipids, Fatty Acids, and Lysine Metabolism Pathways

Interestingly, circulating miRNAs altered in all three comparisons were found to be associated with fatty acid biosynthesis, elongation, metabolism and degradation, as well as lysine degradation.

This is in accordance with data recently reported by our group, which showed a significant association between these pathways and salivary miRNAs of obese pregnant women in the third trimester of pregnancy [25]. These findings may be suggestive of a disarranged fatty acid metabolism, as well as an excessive lysine catabolism in obese pregnancies with or without GDM, which were mediated by miRNA epigenetic alterations. These two independent works suggest that such changes can be detected in different non-invasive-collected body fluids, i.e., maternal saliva and plasma. We also previously reported alterations in metabolome of obese placentas, with markedly lower amounts of lysine and lower levels of Long Chain-Polyunsaturated Fatty Acid (LC-PUFA) derivatives, arachidonic acid, and Docosa-Hexaenoic Acid (DHA), plus significantly increased saturated palmitic acid levels [26].

In addition, miTALOS analysis pointed out an association between OB/GDM(−) vs. NW miRNA pattern and the pathway named “Sterol Regulatory Element-Binding transcription Factor (SREBF) and miR-33 in cholesterol and lipid homeostasis”. hsa-miR-33a-5p is an intronic miRNA located within the gene encoding Sterol Regulatory Element–Binding Factor 2 (SREBF-2), a key transcriptional regulator of cholesterol synthesis, thus modulating the expression of genes involved in cholesterol metabolism [27].

### 4.2. Valine, Leucine, and Isoleucine Metabolism Pathways

Hsa-miR-454-3p (differentially expressed in the comparison OB/GDM(+) vs. NW) was significantly associated with valine, leucine, and isoleucine degradation and biosynthesis pathway.

The branched-chain amino acids (BCAAs) valine, leucine, and isoleucine are essential amino acids, which are primarily obtained from the diet. They have been studied in a number of disorders, notably obesity, metabolic syndrome, liver and kidney disease, muscle disorders, sepsis, cancer, and diabetes mellitus. BCAAs increase rapidly in the systemic circulation after protein intake and are readily available to extrahepatic tissues, in which they stimulate insulin production [28,29].

It has been hypothesized that mammalian Target of Rapamycin Complex 1 (mTORC1) can be activated by BCAA, as well as by insulin, glucose, and cellular ATP availability. This suggests that BCAA overload may cause insulin resistance, together with an increase in BCAA oxidation breakdown products, short-chain acylcarnitines (ACs) [30]. A significant increase in plasma BCAAs and related metabolites was observed in type II diabetes and, notably, in GDM [29]. Indeed, insulin resistance levels in late pregnancy have shown positive correlation with circulating concentrations of BCAAs and ACs. Moreover, also maternal BMI is positively related to circulating concentrations of BCAAs and related short-chain ACs [31]. Because of this, several studies have recently proposed plasma BCAAs as potential biomarkers of GDM [30].

### 4.3. Vitamin B6 Signalling Pathway

The vitamin B6 signalling pathway was associated with OB/GDM(−) vs. NW differentially expressed miRNAs.

Vitamin B6, or pyridoxine, is a water-soluble vitamin found in many foods, including meat, poultry, fish, vegetables, and bananas. It acts as a cofactor in several enzymatic reactions, including glycogen breakdown by glycogen phosphorylase, to obtain glucose for energy production [32]. It is also crucial for one-carbon metabolism related to homocysteine, for provision of methyl groups and sulphur-containing amino acids. It is therefore epigenetically relevant, particularly for methylation of DNA and histones, which impacts the expression of both coding and non-coding genome regions. Indeed, alterations of these extremely complex epigenetic cross-talks could result in deregulated miRNA profiles [33].

Patients with vitamin B6 deficiency show higher circulating levels of systemic markers of oxidative stress and inflammation [34]. Vitamin B6 deficiency has been observed in severely obese subjects, with a negative association between serum vitamin B6 level and BMI [35]. Moreover, overweight or obese individuals receiving long-term vitamin B6 supplementation presented significantly lower weight gain compared to non-supplemented individuals, suggesting that vitamin B6 effects the energetic metabolism that helps control body weight [36].

During early pregnancy, vitamin B6 is, in some cases, indicated to help reduce nausea and vomiting. However, not enough evidence can confirm the clinical benefits of vitamin B6 supplementation in pregnancy. The possible protective role of vitamin B6 is still discussed for pregnancy outcomes, including foetal orofacial clefts, cardiovascular malformations, neurological development, preterm birth, and preeclampsia [37].

### 4.4. mTOR Signalling Pathway

The mammalian Target of Rapamycin (mTOR) signalling pathway was associated with a pattern of miRNAs differentially expressed between OB/GDM(+) and OB/GDM(−).

mTOR pathway acts as a sensor of nutrients, oxygen, and growth factors availability [38]. In particular, mTOR is a key inhibitor of autophagy, especially in relation to oxidative stress and excessive nutrients. The precise relation between mTOR and autophagy in human physiological and pathological pregnancy, particularly in placenta, has yet to be elucidated [39,40].

In obese and diabetic pregnant women, high insulin and leptin activate mTOR signalling in the placenta, promoting mitochondrial function, protein synthesis, and nutrient transport. Indeed, increased expression of placental mTOR has been shown in GDM, especially when associated with obesity. This pathway increases foetal nutrient supply and contributes to oxidative stress, foetal overgrowth, and excessive adiposity in obese and/or diabetic mothers’ offspring [41,42]. The mechanism linking mTOR to oxidative stress and, consequently, to inflammation is based on mTOR overactivation causing increased cellular metabolism, with mitochondrial biogenesis and accumulation of reactive oxygen species (ROS). This involves different tissues and both adult/differentiated and stem cells [43,44]. Concerning high metabolically active cells, mTOR is a particularly relevant signalling pathway in cancer cells. Recent studies have revealed interplays between miRNAs and the mTOR pathway during cancer development [38].

### 4.5. AMPK Signalling Pathway

The miRNA patterns which were significantly different in the comparisons OB/GDM(−) vs. NW and OB/GDM(+) vs. OB/GDM(−) were associated with AMP-activated Protein Kinase (AMPK) signalling.

AMPK is a serine/threonine kinase conserved within eukaryotes. It regulates the whole-body energy homeostasis under stress conditions by sensing the AMP/ATP ratio in almost every cell type. Once activated by falling cellular energy levels, it acts to restore energy homeostasis by turning on catabolic pathways that generate ATP and switching off anabolic pathways and other processes consuming ATP [45,46]. AMPK orchestrates these changes through histone phosphorylation and regulation of proteins involved in nucleosome remodelling. Indeed, during acute metabolic stress, such as fasting or physical exercise, AMPK directly associates with chromatin at promoter regions of genes involved in lipid and glucose metabolism, and activates them to promote energetic molecules availability. At downstream levels, this process enhances mitochondrial biogenesis and function. These observations point to important roles of AMPK as an epigenetic regulator [47].

The dysregulation of AMPK correlates with many different disorders such as cardiovascular disease, diabetes, inflammatory diseases, and cancer. In pregnancy, AMPK is necessary for proper placental differentiation, nutrient transportation, glucose and lipids utilization, and both maternal and foetal energy homeostasis [45,46]. Insulin resistance, obesity-related inflammation and endothelial cell dysfunction affecting adipose, skeletal muscle and placental tissues in maternal obesity and GDM represent possible stressors influencing or being influenced by AMPK activity. Recent data showed reduced AMPK activity in skeletal muscle and adipose tissue of women with GDM [48].

We cannot exclude the contribution of miRNAs in this AMPK deregulation during pregnancy. Interestingly, in our study hsa-let-7b-5p, which is involved in this pathway, resulted as differentially expressed in maternal plasma of OB/GDM(+) vs. OB/GDM(−). Indeed, in obese mouse models, offspring hepatic Let-7 levels were found to be increased, with a consequent decrease in AMPK2α correlating with excessive fat accumulation in hepatocytes [49].

### 4.6. TGF-Beta Signalling Pathway

According to the DIANA-miRPath analysis, miRNA patterns from all comparisons were associated with the Transforming Growth Factor (TGF)-beta signalling pathway. miTALOS analysis confirmed this significant association in OB/GDM(+) vs. NW.

TGF-Beta1 is a widespread anti-inflammatory cytokine with multi-organ effects. It plays active roles in glucose, lipid, amino acid, and redox metabolism, and represents a key cytokine in obesity and insulin resistance. Indeed, elevated TGF-Beta1 levels were associated with glucose intolerance and increased adiposity in women with a prior history of GDM [50,51]. The same pathway emerged from our previous miTALOS analysis of third trimester saliva of obese pregnant women [25]. Significantly decreased TGF-Beta1 gene methylation levels were also reported, supporting TGF-Beta1 upregulation. Therefore, current data reinforce the hypothesis of a complex and epigenetic-regulated inflammatory network in maternal obesity and GDM.

### 4.7. FoxO Signalling Pathway

FOXO (Forkhead box O) proteins represent a subfamily of conserved transcription factors that act as key regulators of longevity downstream of insulin and insulin-like growth factor signalling. In mammals, this subfamily is involved in a wide range of crucial cellular processes regulating oxidative stress, stress resistance, metabolism, cell cycle arrest, and apoptosis [52]. In placenta from obese women, Saben and colleagues observed a significant decrease in total antioxidant capacity, together with increased nuclear FOXO4 localization, which was further significantly associated with activation of the stress-induced c-Jun amino-terminal kinase (JNK). JNK, in turn, plays a pivotal role in regulating cellular oxidative stress in several metabolic conditions such as obesity, insulin resistance, and type II diabetes, reinforcing the evidence that maternal obesity may also be associated with increased oxidative stress in both the placenta and the maternal circulation [53,54]. Importantly, the downstream effectors of both FoxO and JNK signalling include HIF-1α and VEGF-A, which are relevant in the context of maternal obesity, placental hypoxia, inflammation, and oxidative stress [53].

### 4.8. HIF-1 Signalling Pathway

Hsa-miR-454-3p, the single differentially expressed miRNA between OB/GDM(+) and NW women, was significantly associated with HIF-1 signalling pathway.

HIF-1α is a key transcriptional factor activated under hypoxia, playing roles in oxidative stress, inflammation, autophagy, and angiogenesis [55]. It is crucial in early pregnancy, regulating extra villous trophoblast invasion and driving the expression of the pro-angiogenesis factor VEGF. Moreover, it is a known target of Chaperone-Mediated Autophagy [56]. A high glucose concentration impairs the hypoxia-induced HIF-1α activation in human fibroblasts and endothelial cells, suggesting that diabetic hyperglycaemia may result in the loss of cellular adaptation to low oxygen [57,58]. Growing evidence is revealing that the transcriptional activity of HIF-1α, and other HIF pathway members, is regulated at multiple levels by epigenetic mechanisms including DNA methylation, histone methylation/acetylation, and non-coding RNAs, such as miRNAs. The oncologic research showed that specific miRNAs can contribute to HIF-1α stability, promoting VEGF expression and, consequently, angiogenesis and metastasis [59]. Interestingly, analogies between cancer and placenta, in terms of their capacity to proliferate, invade adjacent tissues, generate blood supply, and avoid rejection by the immune system, have been proposed. Some of these functions are regulated right from the HIF pathway [55]. However, to date, only a few studies on placental regulation of HIF signalling by epigenetics and miRNAs have been conducted, mainly in the context of preeclampsia [60].

### 4.9. Insulin Signalling Pathway

The insulin signalling pathway was highlighted by miTALOS analysis, when comparing OB/GDM(−) vs. NW and OB/GDM(+) vs. OB/GDM(−). This pathway did not emerge in OB/GDM(+) vs. NW comparison, possibly because of the presence, in this case, of only one differentially expressed miRNA (i.e., hsa-miR-454-3p).

Colomiere and colleagues reported that in the adipose and skeletal muscle tissue, this pathway plays a role in insulin resistance associated with GDM and maternal obesity [61]. They also showed an alteration of the endogenous expression of insulin signalling components in human term placenta from GDM women versus both non-diabetic obese and healthy normal-weight women [62]. The results of the present study suggest that alterations of the insulin signalling pathway in obese and GDM pregnancies may partially occur through miRNA epigenetic mechanisms.

### 4.10. Germinal Cells, Gametes, and Pluripotency-Related Pathways

According to DIANA-miRPath analysis, some pathways related to germinal cells maturation, oocyte meiosis, and pluripotency of stem cells were common among miRNA patterns from the three comparisons. This is quite surprising, since we analysed maternal plasma at term, while miRNAs are known to finely regulate mechanisms (from gametogenesis to embryogenesis, implantation, and embryo development) within the very first days after conception and during the first trimester of gestation [63,64]. According to the DOHaD hypothesis, it is well known that environmental stressors and a poor intrauterine environment can epigenetically influence not only maternal cells, but also foetal cells. When these issues alter the epigenetic programming of the foetal germ line, these changes can be transmitted across generations in the absence of direct exposure, an effect termed epigenetic transgenerational inheritance [65]. Therefore, it is tempting to speculate that these crucial pathways related to germ cells remain cautiously accessible and non-dormant throughout the gestation period, under the vigilant control of the epigenetic machinery.

### 4.11. Strengths and Limitations

To our knowledge, this is the first study to simultaneously analyse 179 miRNAs in maternal plasma of obese pregnant women with or without GDM. Experiments were performed using a solid Real-time PCR-based method, which included several internal and quality controls. Moreover, the statistical and bioinformatic analyses followed particularly rigorous and stringent rules, ensuring the reliability of the final results. 

A limit of the study is the small number of subjects analysed. Nevertheless, patients were carefully selected and well characterized, presenting homogeneous clinical characteristics in order to avoid possible biases and ensuring the reliability of our results. Notably, only women undergoing elective caesarean section were enrolled, preventing any labour-related variation in miRNAs expression. 

Notably, in the present study, obese women with no comorbidities presented 4 differentially expressed miRNAs compared to NW and 14 compared to obese women with GDM, while the latter presented only 1 miRNA with a significant decrease compared to NW. This may be due to the low number of patients. However, another issue to consider is that in clinical practice, obese pregnant women presenting GDM undergo a deeper examination, with a multidisciplinary management from different professionals (gynaecologist, diabetologist, nutritionist, etc.) and more frequent checking of ambulatory controls. This can help the patient comply with an appropriate lifestyle, which is also suggested, in the present population study, by the lower gestational weight gain in OB/GDM(+) compared to OB/GDM(−) and to NW.

In addition, in the present study, normal weight women with GDM were not included, due to the low number of cases admitted in the enrolling Hospital. Indeed, GDM prevalence is rated based on BMI categories, decreasing with BMI from 5.5–11.5% in obese to 2.3% in normal weight [66]. Further research analysing a larger population, possibly involving in vitro models, is therefore needed to comprehensively frame possible molecular mechanisms highlighting miRNA-specific functions during pregnancy and in the context of gestational metabolic diseases. 

Another topic that should be considered is that maternal lifestyle and dietary habits might impact miRNA expression. Future studies investigating maternal nutritional condition and physical activity/inactivity with the use of specific questionnaires will allow us to integrate the present data in order to outline a deeper picture of the complex interactions among maternal habits, metabolism, epigenetics, and the intrauterine environment.

Finally, the identification of the origin of circulating miRNAs, which might be generated by both maternal and foetal organs and the placenta, could also add important information on the pathogenesis of the epigenetic alterations in these pregnancies.

## 5. Conclusions

This pilot study preliminarily reported a pattern of differentially expressed miRNAs in the maternal plasma of obese women at term of pregnancy, in the presence or absence of gestational diabetes mellitus. Thus, it shed light on the complex epigenetic network regulating many metabolic pathways in both the mother and the foeto-placental unit during pregnancy. Indeed, miRNAs in the maternal plasma are also the result of the foeto-placental release of molecules into the maternal circulation.

Several pathways have been associated with miRNAs that presented altered expression in obese women, mostly involving cell and energy metabolism, the redox balance and inflammation signalling, thus highlighting the strong impact that alterations of the epigenetic regulation can provoke in these pregnancies.

The results obtained in this pilot study lay the foundation for the investigation of specific miRNAs expression during early gestation, as well as offering potential identification of early non-invasive biomarkers beneficial for both monitoring the pregnancy course and identifying possible gestational complications.

Pregnancy is a window of susceptibility and plasticity, which might be used as an opportunity. Future interventional studies investigating the impact of lifestyle and dietary changes on epigenetics and miRNA patterns in the mother, the foetus, and the placenta might open up new perspectives for possible non-pharmacological therapies used to prevent negative outcomes in maternal obesity and GDM.

The present results enrich the current knowledge of the epigenetic landscape during pregnancy, representing a starting point for future research using miRNAs as possible biomarkers and therapeutic agents or targets for the diagnosis and treatment of pregnancy pathologies.

## Figures and Tables

**Table 1:** Maternal and foetal characteristics of the population.

	NW (*n* = 7)	OB/GDM(−) (*n* = 6)	OB/GDM(+) (*n* = 6)
Age ^1^ [years]	32.29 ± 3.64	33.33 ± 6.83	33.33 ± 5.01
Pre-pregnancy BMI ^2^ [Kg/m^2^]	21.57 ± 2.37	32.55 ± 2.23 *	34.63 ± 2.87 **
OGTT time 0 ^1^ [mg/dL]	83.25 ± 1.26	84.60 ± 6.23	86.20 ± 15.32
OGTT time 60^min 1^ [mg/dL]	128.00 ± 31.86	125.40 ± 36.31	158.60 ± 33.45
OGTT time 120^min 1^ [mg/dL]	127.74 ± 51.50	109.40 ± 30.18	162.60 ± 30.74
GWG ^1^ [Kg]	10.86 ± 2.48	8.08 ± 3.96	5.60 ± 7.30
Gestational age ^2^ [weeks]	39.20 ± 0.24	39.00 ± 0.42	39.08 ± 0.17
Foetal weight ^1^ [g]	3345.71 ± 135.54	3415.00 ± 386.51	3475.83 ± 358.70
Foetal weight centile ^1^	51.00 ± 17.59	53.17 ± 28.97	61.17 ± 24.72
Foetal sex [%]	F: 43; M: 57	F: 17; M: 83	F: 50; M: 50

Data are presented as average ± standard deviation and analysed according to their distribution with ^1^ One-way ANOVA (Tukey’s HSD as post hoc test with Bonferroni correction) or ^2^ Kruskal–Wallis tests (pairwise comparison as post hoc test with Bonferroni correction). * *p* < 0.05, ** *p* < 0.01 vs. NW. Foetal sex distribution was analysed with Fisher’s Exact Probability Test: non-significant. [BMI: Body Mass Index; OGTT: Oral Glucose Tolerance Test; GWG: Gestational Weight Gain; F: female; M: male].

**Table 2:** Differentially expressed miRNAs in maternal circulation.

(A) OB/GDM(−) vs. NW		
miRNA ID	Fold Regulation	*p*-Value
hsa-miR-27a-3p	2.13	0.0016
hsa-miR-324-5p	2.29	0.0018
hsa-miR-33a-5p	3.38	0.0042
hsa-miR-186-5p	−2.26	0.0155
**(B) OB/GDM(+) vs. NW**		
**miRNA ID**	**Fold Regulation**	***p*-value**
hsa-miR-454-3p	−2.32	0.0216
**(C) OB/GDM(+) vs. OB/GDM(−)**		
**miRNA ID**	**Fold Regulation**	***p*-value**
hsa-miR-186-5p	2.13	0.0065
hsa-miR-320d	2.18	0.0148
hsa-miR-2110	2.04	0.0196
hsa-let-7b-5p	2.09	0.0260
hsa-miR-574-3p	2.45	0.0266
hsa-miR-320c	2.26	0.0377
hsa-miR-324-5p	−2.75	0.0023
hsa-miR-142-3p	−3.03	0.0115
hsa-miR-33a-5p	−4.09	0.0144
hsa-miR-21-5p	−12.16	0.0162
hsa-miR-27a-3p	−2.02	0.0186
hsa-let-7f-5p	−3.45	0.0294
hsa-miR-30e-3p	−4.57	0.0403
hsa-miR-339-5p	−2.46	0.0467

For each panel, upregulated miRNAs are listed first and downregulated miRNAs are listed later (a double line separates the two sections); each section is ordered by increasing *p*-value (decreasing statistical significance). Statistical significance: *p*-value < 0.05; Fold Regulation > |2|, with positive values (>2) indicating upregulation and negative values (<−2) indicating downregulation in “case” group vs. related “control”. Data were analysed using GeneGlobe (Qiagen).

**Table 3:** Selected pathways coherent with the context of this study, from the union analysis performed with DIANA–miRPath v.3, for each comparison: (A) OB/GDM(−) vs. NW, (B) OB/GDM(+) vs. NW, (C) OB/GDM(+) vs. OB/GDM(−).

(A) OB/GDM(−) vs. NW			
Pathway Name	Pathway Information	*p*-Value	miRNAs
Fatty acid biosynthesis	Creation of fatty acids from acetyl-CoA and NADPH through fatty acid synthases.	3.2455 × e^−12^	hsa-miR-27a-3p
ECM (ExtraCellular Matrix)-receptor interaction	Complex mixture of structural and functional macromolecules with important roles in cell, tissue and organ morphogenesis, structure, and function.	1.6770 × e^−8^	hsa-miR-27a-3p
AMPK (AMP-activated Protein Kinase) signalling pathway	Sensor of cellular energy status.	3.5242 × e^−5^	hsa-miR-27a-3p hsa-miR-186-5p
TGF-beta (Transforming Growth Factor-beta) signalling pathway	Regulation of cellular functions such as proliferation, apoptosis, differentiation, and migration	3.7782 × e^−5^	hsa-miR-27a-3p hsa-miR-186-5p
Lysine degradation	Amino acid breakdown mainly taking place in hepatocytes’ mitochondria.	6.9088 × e^−5^	hsa-miR-27a-3p hsa-miR-33a-5p hsa-miR-186-5p
Oocyte meiosis	Maturation of female gametes.	0.000417	hsa-miR-27a-3p hsa-miR-33a-5p
Thyroid hormone signalling pathway	Thyroid hormones triiodothyronine (T3) and thyroxine (T4) are important regulators of growth, development, and metabolism.	0.0016	hsa-miR-27a-3p
FoxO (Forkhead box O) signalling pathway	Transcription factors regulating apoptosis, cell-cycle control, glucose metabolism, oxidative stress resistance, and longevity.	0.003443	hsa-miR-27a-3p
Signalling pathways regulating pluripotency of stem cells	Pluripotent stem cells (PSCs) are self-renewal cells with the potential to generate all cell types of the three germinal layers.	0.030599	hsa-miR-27a-3p
Progesterone-mediated oocyte maturation	Insulin/IGF-1 or progesterone exposure induces maturation of the oocyte into a mature, fertilizable egg.	0.037975	hsa-miR-27a-3p hsa-miR-186-5p
Vitamin B6 metabolism	Coenzyme in amino acid, glucose, and lipid metabolism.	0.040007	hsa-miR-186-5p
**(B) OB/GDM(+) vs. NW**			
**Pathway name**	**Pathway information**	***p*-value**	**miRNAs**
Fatty acid elongation	Part of the anabolic processes generating and modifying fatty acids.	1.3166 × e^−11^	hsa-miR-454-3p
Fatty acid degradation	Fatty acids breakdown into their metabolites, and finally acetyl-CoA.	1.3726 × e^−7^	hsa-miR-454-3p
Lysine degradation	Amino acid breakdown mainly taking place in hepatocytes’ mitochondria.	8.4918 × e^−5^	hsa-miR-454-3p
Fatty acid metabolism	Catabolic and anabolic processes involving fatty acids.	8.4918 × e^−5^	hsa-miR-454-3p
Signalling pathways regulating pluripotency of stem cells	Pluripotent stem cells (PSCs) are self-renewal cells with the potential to generate all cell types of the three germinal layers.	0.009942	hsa-miR-454-3p
TGF-beta (Transforming Growth Factor-beta) signalling pathway	Regulation of cellular functions such as proliferation, apoptosis, differentiation, and migration	0.010679	hsa-miR-454-3p
FoxO (Forkhead box O) signalling pathway	Transcription factors regulating apoptosis, cell-cycle control, glucose metabolism, oxidative stress resistance, and longevity.	0.037202	hsa-miR-454-3p
Valine, leucine and isoleucine (BCAA) degradation	Breakdown of the branched-chain amino acids (BCAA).	0.038549	hsa-miR-454-3p
HIF-1 (Hypoxia-inducible factor 1) signalling pathway	Transcription factor, master regulator of oxygen homeostasis. Involvement in autophagy, inflammation, and oxidative stress.	0.043120	hsa-miR-454-3p
Valine, leucine and isoleucine biosynthesis	Enzymatic process generating branched-chain amino acids (BCAA).	0.049787	hsa-miR-454-3p
**(C) OB/GDM(+) vs. OB/GDM(−)**			
**Pathway name**	**Pathway information**	***p*-value**	**miRNAs**
Fatty acid biosynthesis	Creation of fatty acids from acetyl-CoA and NADPH through fatty acid synthases.	<1 × e^−325^	hsa-miR-2110 hsa-miR-27a-3p
ECM (ExtraCellular Matrix)-receptor interaction	Complex mixture of structural and functional macromolecules with important roles in cell, tissue, and organ morphogenesis, structure, and function.	<1 × e^−325^	hsa-miR-27a-3p hsa-let-7f-5p hsa-miR-30e-3p
Lysine degradation	Amino acid breakdown mainly taking place in hepatocytes’ mitochondria.	<1 × e^−325^	hsa-miR-186-5p hsa-miR-2110 hsa-let-7b-5p hsa-miR-574-3p hsa-miR-142-3p hsa-miR-33a-5p hsa-miR-21-5p hsa-miR-27a-3p hsa-let-7f-5p hsa-miR-30e-3p hsa-miR-339-5p
TGF-beta (Transforming Growth Factor-beta) signalling pathway	Regulation of cellular functions such as proliferation, apoptosis, differentiation, and migration.	9.0845 × e^−8^	hsa-miR-186-5p hsa-let-7b-5p hsa-miR-320c hsa-miR-27a-3p hsa-miR-30e-3p
FoxO (Forkhead box O) signalling pathway	Transcription factors regulating apoptosis, cell-cycle control, glucose metabolism, oxidative stress resistance, and longevity.	3.6540 × e^−6^	hsa-let-7b-5p hsa-miR-21-5p hsa-miR-27a-3p hsa-let-7f-5p hsa-miR-30e-3p
Thyroid hormone signalling pathway	Thyroid hormones triiodothyronine (T3) and thyroxine (T4) are important regulators of growth, development, and metabolism.	3.9364 × e^−6^	hsa-let-7b-5p hsa-miR-21-5p hsa-miR-27a-3p hsa-let-7f-5p
Fatty acid metabolism	Catabolic and anabolic processes involving fatty acids.	1.9484 × e^−5^	hsa-miR-2110 hsa-miR-21-5p hsa-miR-27a-3p
Oocyte meiosis	Maturation of female gametes.	7.3982 × e^−5^	hsa-let-7b-5p hsa-miR-33a-5p hsa-miR-27a-3p hsa-let-7f-5p
AMPK (AMP-activated Protein Kinase) signalling pathway	Sensor of cellular energy status.	0.004058	hsa-miR-186-5p hsa-let-7b-5p hsa-miR-27a-3p

Pathway information and description have been found on the online repository https://www.genome.jp/ accessed on 25 July 2022, in particular in the section KEGG PATHWAY Database; *p*-value indicates the force of association between the pathway and specific miRNAs (indicated in the last column); pathways are listed in order of increasing *p*-value (decreasing statistical significance).

## Data Availability

The data presented in this study are available in the article or Supplementary Material.

## Supplementary Materials

The following supporting information can be downloaded at: https://www.mdpi.com/article/10.3390/antiox12020515/s1, Supplementary Table S1. Results of the union analysis performed with DIANA–miRPath v.3.
