# Supplementary material for: Characterization of Maternal Circulating MicroRNAs in Obese Pregnancies and Gestational Diabetes Mellitus
Source: Antioxidants (Basel). 2023 Feb 17;12(2):515. doi: 10.3390/antiox12020515 (PMC9952647; doi:10.3390/antiox12020515)
Supplement: Supplementary file 1 [file antioxidants-12-00515-s001.zip › antioxidants-2219146-supplementary.pdf]

Supplementary Table S1 | Results of the union analysis performed with DIANA–miRPath v.3

Pathway Enrichment Analysis results (DIANA-miRPath):		
**A) OB/GDM(−) vs NW**	**B) OB/GDM(+) vs NW**	**C) OB/GDM(+) vs OB/GDM(−)**
1. Prion diseases<br>2. Viral carcinogenesis<br>3. Hippo signalling pathway<br>**4. Fatty acid biosynthesis**<br>**5. ECM-receptor interaction**<br>6. Protein processing in endoplasmic reticulum<br>7. Proteoglycans in cancer<br>8. Adherens junction<br>9. Bacterial invasion of epithelial cells<br>**10. AMPK signalling pathway**<br>11. Chronic myeloid leukemia<br>**12. TGF-beta signalling pathway**<br>**13. Lysine degradation**<br>14. Mucin type O-Glycan biosynthesis<br>15. Cell cycle<br>16. p53 signalling pathway<br>**17. Oocyte meiosis**<br>18. Glioma<br>19. Neurotrophin signalling pathway<br>20. Thyroid cancer<br>**21. Thyroid hormone signalling pathway**<br>**22. FoxO signalling pathway**<br>23. Colorectal cancer<br>24. Pathways in cancer<br>25. Prostate cancer<br>26. Transcriptional misregulation in cancer<br>27. Axon guidance<br>28. Renal cell carcinoma<br>29. Shigellosis<br>30. Focal adhesion<br>31. Hepatitis B<br>**32. Signalling pathways regulating pluripotency of stem cells**<br>33. Endometrial cancer<br>**34. Progesterone-mediated oocyte maturation**<br>**35. Vitamin B6 metabolism**	**1. Fatty acid elongation**<br>**2. Fatty acid degradation**<br>**3. Lysine degradation**<br>**4. Fatty acid metabolism**<br>**5. Signalling pathways regulating pluripotency of stem cells**<br>**6. TGF-beta signalling pathway**<br>**7. FoxO signalling pathway**<br>**8. Valine, leucine and isoleucine degradation**<br>9. Bacterial invasion of epithelial cells<br>**10. HIF-1 signalling pathway**<br>11. RNA transport<br>12. Ubiquitin mediated proteolysis<br>13. Glioma<br>14. Proteoglycans in cancer<br>**15. Valine, leucine and isoleucine biosynthesis**	**1. Fatty acid biosynthesis**<br>**2. ECM-receptor interaction**<br>3. Prion diseases<br>4. Viral carcinogenesis<br>5. Hippo signalling pathway<br>**6. Lysine degradation**<br>7. Proteoglycans in cancer<br>8. Cell cycle<br>9. Adherens junction<br>10. Chronic myeloid leukemia<br>**11. TGF-beta signalling pathway**<br>12. Hepatitis B<br>13. Glioma<br>14. p53 signalling pathway<br>**15. FoxO signalling pathway**<br>**16. Thyroid hormone signalling pathway**<br>17. Protein processing in endoplasmic reticulum<br>18. Bacterial invasion of epithelial cells<br>**19. Fatty acid metabolism**<br>20. Pathways in cancer<br>21. Endocytosis<br>22. Colorectal cancer<br>**23. Oocyte meiosis**<br>24. Thyroid cancer<br>25. Transcriptional misregulation in cancer<br>26. Prostate cancer<br>27. Epstein-Barr virus infection<br>28. Small cell lung cancer<br>29. Mucin type O-Glycan biosynthesis<br>**30. AMPK signalling pathway**<br>31. MAPK signalling pathway<br>32. Pancreatic cancer<br>33. Neurotrophin signalling pathway<br>34. Melanoma<br>35. Renal cell carcinoma<br>36. Endometrial cancer<br>37. Steroid biosynthesis<br>38. Bladder cancer

All listed pathways resulted significantly associated (force of association: *p*-value <0.05) with the related miRNAs' cluster, for each comparison **A)** OB/GDM(−) vs NW, **B)** OB/GDM(+) vs NW, **C)** OB/GDM(+) vs OB/GDM(−) (refer to Table 2 for the miRNA clusters). Pathways are ordered by descending statistical significance, p-values not showed. Pathways considered to be the most interesting ones according to the context of maternal obesity, GDM, pregnancy, inflammation have been marked with bold font.
